# Functional role for Taz during hindbrain ventricle morphogenesis

**DOI:** 10.1371/journal.pone.0313262

**Published:** 2025-03-13

**Authors:** Renée Dicipulo, Lyndsay G. Selland, Rowan G. Carpenter, Andrew J. Waskiewicz

**Affiliations:** 1 Department of Biological Sciences, University of Alberta, Edmonton, Alberta, Canada; 2 Women & Children’s Health Research Institute, University of Alberta, Edmonton, Canada; Laboratoire de Biologie du Développement de Villefranche-sur-Mer, FRANCE

## Abstract

The brain ventricle system, composed of the ventricular cavities and the cerebral spinal fluid within, performs critical functions including circulation of nutrients, removal of wastes, and cushioning of neural tissues. Development of the hindbrain ventricle requires a series of factors that coordinate its initial formation and subsequent inflation. Previous work has demonstrated that the transcriptional co-activator Taz (also known as WW domain-containing transcription regulator protein 1, Wwtr1), a component of Hippo signalling, is active at hindbrain rhombomere boundaries where it is regulated by mechanotransduction and promotes proliferation. Here, we demonstrate that Taz is also a critical regulator of hindbrain ventricle development. Zebrafish embryos that lack Taz protein fail to undergo initial midline separation of the hindbrain ventricle. Furthermore, the ventricle phenotype is a result of disorganized cytoskeletal F-actin and apicobasal polarity components. In addition, we have demonstrated that the hindbrain rhombomere boundaries are a location of active Wnt-Hippo crosstalk. Through our work, we propose a model where Taz protein is stabilized at rhombomere boundaries and promotes proper cell polarity necessary for formation of the brain ventricle.

## Introduction

During embryonic development, the brain ventricle system (BVS; composed of a series of cavities and connecting channels filled with cerebral spinal fluid) undergoes highly orchestrated morphological changes, requiring coordinated cellular movement, alterations to cell adhesion, and proliferation [1–5]. The BVS is necessary for the circulation of nutrients, removal of wastes, and cushioning and protection of the brain [2,6]. Aberrant BVS formation results in a disturbance of cerebrospinal fluid (CSF) flow and is associated with neurodegenerative disease [3,7]

The BVS has been studied across a wide spectrum of vertebrate animal models – from the components within the CSF to the morphological stages of BVS development. Research in chick and zebrafish have elucidated the main morphological processes evident in its development. In teleost fish, the formation of the neural tube begins with a neuroepithelial flat sheet (termed neural plate), which when columnarized, forms a neural keel [8–10]. Subsequently, this neural keel becomes a neural rod, which then opens on the luminal side, resulting in the neural tube [11,12]. This structure then inflates by the release of cerebral spinal fluid and transient sealing of the neural tube [1].

Concurrent to the formation of BVS, the hindbrain tissue, initially a long sheet of undifferentiated cells, compartmentalize into metameric units known as rhombomeres [13]. Cells within rhombomeres are lineage-restricted and this segmentation prevents the mixing of cell lineages; this early restriction of cells is integral for the later organization of differentiated neurons in the hindbrain [13–16]

Mirroring the complexity of its development, numerous signalling pathways have been identified as necessary components for proper hindbrain and hindbrain ventricle development [17–19]. In the hindbrain, Wnt, Notch, retinoic acid (RA) and fibroblast growth factor (FGF) signaling pathways are required for proper segmentation [20–24]. FGF and RA are known to regulate segment-specific gene expression whereas Wnt and Notch are active at hindbrain rhombomere segment boundaries [25–27]. Our work proposes the necessity of Hippo signalling in hindbrain and subsequent brain ventricle development.

The Hippo signaling pathway has been shown to regulate the development of organs via restriction of organ size through mechanisms such as cell proliferation and apoptosis [28,29]. As a regulator of tissue growth, several upstream signals coordinate the activation of the Hippo signaling pathway, with inputs including cell density, cell-cell contact, mechanical force, growth factors and GPCR signaling [30,31]. The Hippo signalling pathway consists of a core kinase cascade that when active, results in the phosphorylation and inactivation of Yap and Taz, two transcription co-factors [32]. In the absence of Hippo signaling (when the core kinases are inactive), Yap and Taz translocate to the nucleus and activate gene transcription via interaction with TEAD transcription factors. Within the hindbrain, recent work has demonstrated that the Hippo signalling system acts as sensors of mechanotransducive forces and this nexus of signaling is necessary for later hindbrain segmentation and maintenance of the boundaries that restrict cell lineage between rhombomeres [17,33]. In addition, Taz/Yap-TEAD activity has been shown to regulate the proliferative capacity of boundary cells [17] and Taz-Tead1a mediated transcription results in sharp boundary formation and proper boundary marker expression [33]. While the processes that require Taz activity at rhombomere boundaries have been identified, the functional phenotypes relating to BVS development have not been explored.

In addition to the known role for Hippo signalling at rhombomere boundaries, components of the canonical Wnt signaling pathway are also localized to boundary cells. Wnt regulates processes such as cell proliferation, cell polarity, cell fate determination as well as tissue homeostasis [34]. Wnt1 has been shown to be necessary for neurogenesis in zebrafish, where Wnt promotes proneural and *delta* gene expression within rhombomeres and a growing body of evidence suggests that the Wnt pathway interacts with Taz/Yap [35,36].

Here, we demonstrate the novel discovery that Taz is required for early hindbrain ventricle development. Specifically, we show that *taz-/-* mutants have reduced hindbrain ventricles with defects apparent from the earliest steps of midline separation. Additionally, we show that *taz* mRNA is expressed in a striated manner and this striation is still apparent when looking at Taz protein. Through pharmacological manipulation, we provide evidence that Wnt signalling is a key regulator of the spatial organization of *taz* mRNA and Taz protein. We also identify an interaction between Taz and Axin1, a scaffolding component of the β-catenin destruction complex. In addition, *taz-/-* mutants display disorganization of apicobasal polarity components at the presumptive lumen in locations where the rhombic lips fail to separate. Taken together, our results support a model whereby Wnt and Hippo pathways coordinate to regulate the initial morphogenesis of the hindbrain and hindbrain ventricle.

## Results

### taz mutants display midline separation defects

**Fig 1:**
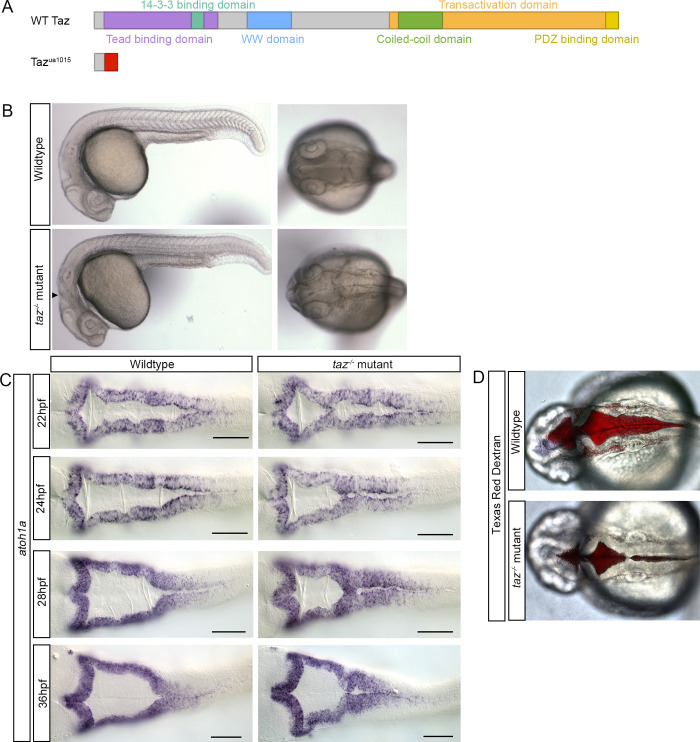
*taz*^-/-^ mutants show midline separation defects. (A) Predicted transcripts of wild-type (top) and the TALEN generated *taz*^ua1015^ mutant allele (bottom). The zebrafish taz^ua1015^ mutant allele contains a 29 bp deletion, 25 bp downstream from the start codon, which results in a frameshift mutation and loss of all functional domains. (B) Microscopic analysis of embryonic phenotypes. Wild-type (top) and *taz-/-* mutant embryos (bottom) were analyzed at 24 hpf, shown at both lateral and dorsal views. Consistently, *taz-/-* mutants display disruptions to hindbrain morphology (lateral view, arrowhead) as well as failure of the brain ventricle midline separation (dorsal view, arrowhead). (C) *atoh1a in situ* hybridization on wild-type and *taz-/-* mutant embryos from 22-36 hpf were performed to visualize ventricle perturbations over the course of development. (D) Texas Red Dextran injections were performed into the ventricle of wild-type and *taz-/-* mutant embryos at 24 hpf to visualize extent of ventricle defects in live animals. Scale bar =  100 µm.

We first sought to characterize Taz/Wwtr1 (hereafter abbreviated as Taz) in the hindbrain ventricle by analyzing embryos that lack Taz function. To generate *taz* mutants, we designed transcription activator-like effector nucleases (TALENs) to interrupt the coding sequence prior to conserved domains. Zebrafish *taz*^*ua1015*^ mutants encode a 29 nucleotide (nt) deletion 25 nt downstream from the predicted start codon (*wwtr1*-201.c.25-54del), resulting in a frameshift mutation that results in a stop codon 12 amino acids downstream. (p.Ile9LysfsX12; Fig 1A; hereafter *taz* mutant homozygotes will be denoted as *taz-/-* mutants).

We examined the morphology of homozygous *taz-/-* mutant embryos at 24 hours post fertilization (hpf) and found that compared to wild-type embryos, *taz-/-* mutant embryos consistently display alterations to the morphology of the central nervous system (Fig 1B). This includes loss of mid-hindbrain boundary definition (dorsal and lateral views), reduced hindbrain rhombomere segment definitions and diminished height of brain ventricle (Fig 1B). In contrast, development of posterior structures, such as somites and tail fin appear normal in *taz-/-* mutants.

Injection of wild-type *taz* mRNA showed an increase in the hindbrain ventricle size in *taz-/-* mutants (Fig 1A) and of the truncated *taz* mRNAs, the construct encoding a deletion of only the PDZ binding domain (the ΔPDZ construct) was able to significantly rescue the ventricle phenotype of *taz*^-/-^ mutants, as GFP injected *taz-/-* mutants had significantly smaller ventricles compared to ΔPDZ construct injected animals (p = 0.00005; S1 Fig C-C’). Since all deletions aside from the PDZ domain construct lack the transactivation domain, we also provide evidence that the transactivation domain is required for brain ventricle morphogenesis.

**Fig 2:**
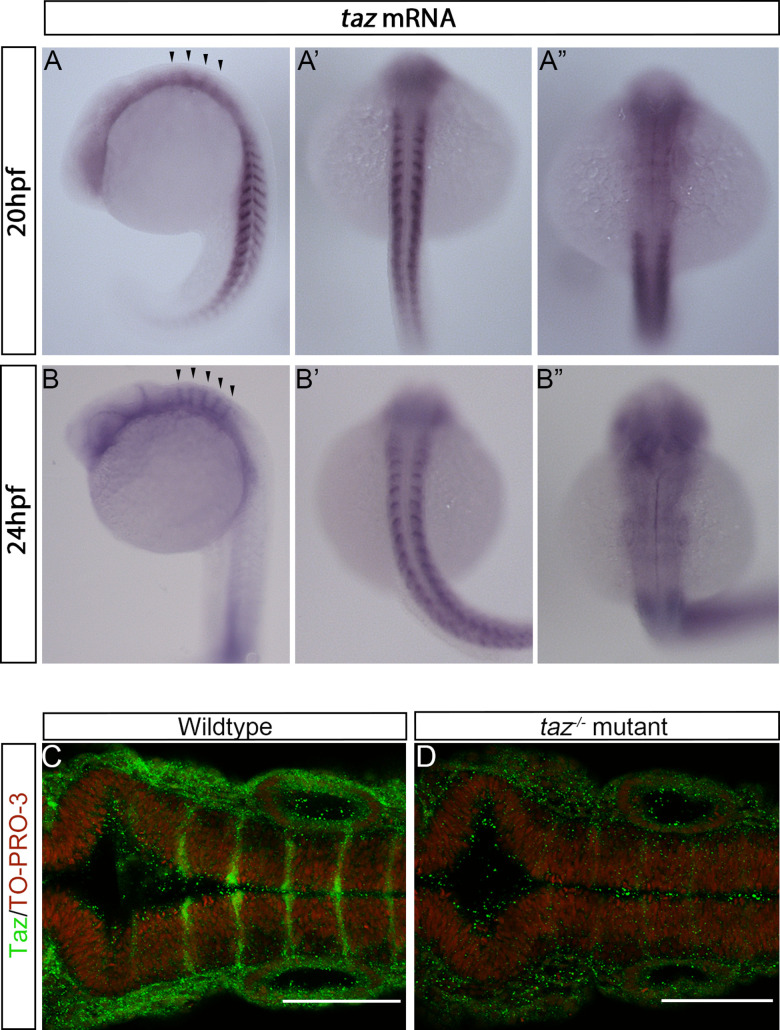
*taz* mRNA and Taz protein are seen in the hindbrain during BVS development. (A-A”). *In situ* hybridization of wild-type embryos stage 20 hpf show *taz* expression throughout the embryo, from the trunk and tail to broadly in the rostral region, shown both laterally (A) and dorsally (A’, A”). (A’) Dorsal view of the embryo showing *taz* expression in the somite region. (A”) Dorsal view of the embryo hindbrain showing *taz* expression is enriched at rhombomere boundaries. (B-B”). At 24 hpf, broad expression of *taz* in the anterior and in the trunk is still seen; *taz* mRNA enrichment at rhombomere boundaries also persists. (C) Taz protein is localized to rhombomere boundaries in wild-type 24 hpf embryos whereas (D) *taz-/-* mutants display only background signal.

Brain ventricle mutants can be separated into two major categories based on which process is affected: 1) initial brain shaping and inflation where the ventricles do not take the correct form, which occurs between 18 and 24 hpf; and 2) later ventricle expansion, where the ventricle is reduced in size which occurs from 24 to 30 hpf [37]. Additionally, in other BVS mutant models, disruption of factors can affect the initial stage of brain morphogenesis but during ventricle expansion, the increased luminal pressure resolves the phenotype such that they appear like wild-type animals in later embryonic stages, this resolution is thus indicative of severity of the mutant. Therefore, we sought to determine at which phase of brain ventricle morphogenesis Taz functions, and if the ventricle phenotype persists beyond 24 hpf by examining the ventricle in greater detail during initial BVS shaping and ventricle expansion (from 22 hpf to 36 hpf). To visualize ventricle defects, we utilized in situ hybridization with *atoh1a* (*atonal bHLH transcription factor 1a*), which is expressed in the upper and lower rhombic lips to outline the ventricle [38]. Mutant *taz-/-* embryos at 22 hpf have hindbrain ventricles where the midline fails to open uniformly. This contrasts with other published brain ventricle mutants, where the midline completely fails to separate [37]. Further examination of older *taz*^-/-^ mutants shows that this phenotype persists until 36 hpf (Fig 1C). Additionally, in scoring *taz*^ + /-^ heterozygotes for the midline separation phenotype, 37.5% of 22 hpf *taz*^ + /-^ heterozygotes show a reduced ventricle size but this phenotype failed to persist post 24 hpf, suggesting that *taz*^ + /-^ heterozygotes may have a delay in brain ventricle formation that resolves post 24hpf (Table 1). To quantify the reduction in ventricle size, we measured the size of the ventricle of wild-type and *taz-/-* mutant samples. We observed a statistically significant reduction in size at each stage from 22-36 hpf (S2 Fig 2, p < 0.001).

**Table 1:** Penetrance of hindbrain midline defect across genotypes.

Age	22hpf	24hpf	28hpf	36hpf	48hpf
*taz-/-*	8/8	11/12	9/11	10/14	1/4
*taz* ^+^ ^*/-*^	9/24	1/24	0/26	0/23	0/14
*taz* ^+ / +^	1/11	0/12	0/6	0/6	0/5

Our results suggest that Taz has a role in early brain ventricle morphogenesis and lack of the phenotype resolving after brain ventricle inflation at 24 hpf indicates that the phenotype is severe.

Analyses of *taz-/-* mutant brain ventricle size via *atoh1a in situ* provided insight to ventricle size changes over time. To confirm defects in brain ventricle development were not due to artifacts from the fixation, dissection and mounting of hindbrain samples, Texas Red Dextran was injected into the cerebrospinal fluid of live embryos at 24 hpf. We injected dye into the cerebrospinal fluid and examined the phenotype at 24 hpf [39]. Compared to wild-type animals, the *taz*^-/-^ mutants display ventricle lips that fail to separate at the midline, resulting in aberrant apposition and reduced ventricle size (Fig 1D).

In vertebrates, the Hippo signaling pathway contains another downstream transcriptional co-activator, the Yes1 associated transcriptional regulator, Yap1 (yes-associated protein 1; which will be denoted as Yap). Because we observed brain ventricle phenotypes when there was loss of Taz, we wanted to see if Yap was also involved in brain ventricle morphogenesis. To generate a *yap* mutant allele, we utilized CRISPR-Cas9 technology. The *yap*^*ua1027*^ allele contains a 14 nt deletion 604 nt downstream from the start codon, resulting in a predicted frameshift mutation, disrupting several domains including the WW1 and WW2 domain (*yap-*201.c.604-618del, p.Asp111AlaX11). In *yap-/-* homozygous mutants, the ventricle size was comparable to wild-type animals at both 22 hpf and 24 hpf (S3 Fig A). This suggests that Taz is the principal Hippo pathway component in regulation of BVS development. We were unable to examine the phenotype of *taz-/-;yap-/-* double mutants, as embryos that lack both genes arrest in development during early somitogenesis similar to other *taz-/-;yap-/-* double mutants published previously [40].

Hindbrain boundaries show *taz* mRNA expression and Taz protein localization

To identify which cells are involved in Taz-dependent BVS development, we performed whole mount *in situ* hybridization for *taz* mRNA at developmental stages during the initial phases of brain ventricle midline separation. *taz* mRNA expression is seen at early stages in BVS development, with mild expression in neural tissues at 16 hpf and this diffuse expression persists to 18 hpf (S4 Fig A-B”). In zebrafish embryos staged 20 hpf, *taz is* expressed in neural tissues and in the somite myoseptum (Fig 2A-A’). Interestingly, discernible stripes of enriched *taz* mRNA expression are seen in the hindbrain (Fig 2A, 2A”) and this pattern of expression is maintained post 20 hpf (S4 Fig 4D-D”). At 24 hpf, *taz* expression in the hindbrain is more pronounced, and this expression aligns with the boundaries of structures in the hindbrain known as rhombomeres (Fig 2B-B”). Additionally, *taz* expression is seen broadly in the anterior nervous system of wild-type embryos, and is seen more strongly in the trunk somite region, consistent with other reports (Fig 2B-B”, [41]). However, at 28 hpf and 48 hpf, *taz* expression is markedly reduced (S4 Fig F-G’).

**Fig 3:**
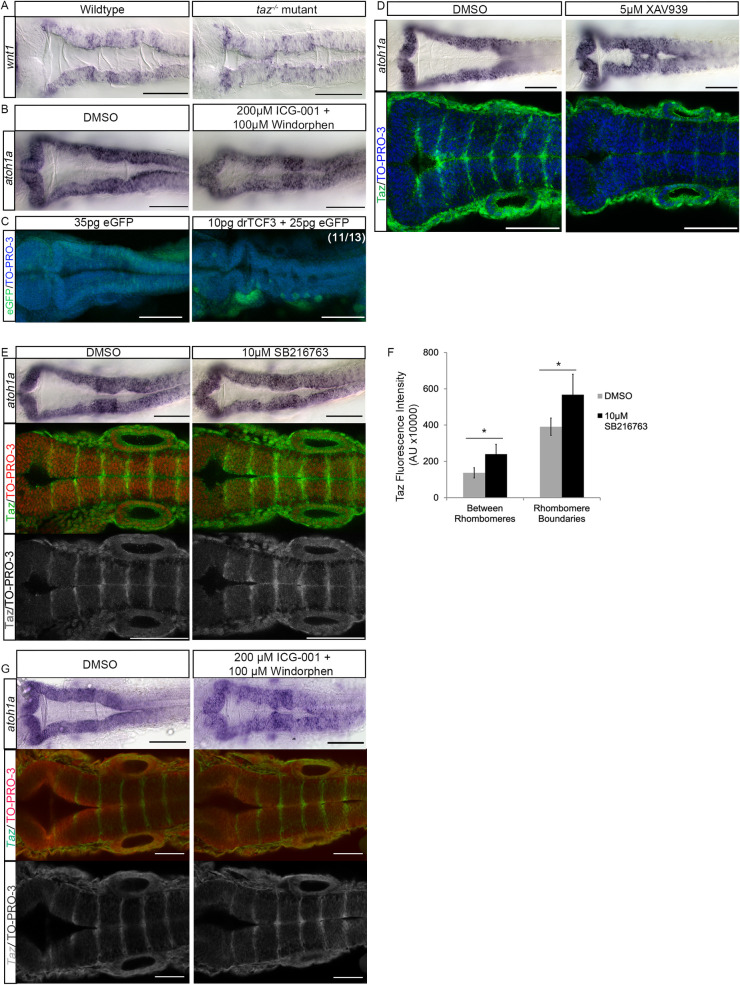
Wnt components are modified in *taz*^-/-^ mutants and inhibition of β-catenin mediated transcription results in reduced ventricle size. (A) *in situ* hybridization of *wnt1* shows that in wild-type animals *wnt1* expression is seen as striations along the hindbrain ventricle. In *taz-/-* mutants, this expression is disorganized. (B) Wild-type embryos were treated with 200 µM ICG-001 and 100 µM Windorphen to inhibit β-catenin mediated transcription. Compared to DMSO controls, treated embryos show reductions in ventricle size. (C) Similarly, inhibition of β-catenin mediated transcription via injection of 10 pg of a drTCF3 resulted in a reduced ventricle size compared to controls injected with eGFP alone. Cell nuclei labelled with TO-PRO-3. (D) Compared to wild-type animals, treatment with 5 µM XAV939 reduced ventricle size as visualized using *atoh1a in situ* (top). Additionally, XAV939 treated animals show a reduction of Taz protein localization at rhombomere boundaries (bottom). Cell nuclei labelled with TO-PRO-3. (E) Animals treated with 10 µM SB216763 show little change to ventricle shape and size compared to wild type animals, visualized using *atoh1a in situ* (top). However, SB216763 animals show an increase in Taz signal throughout the rhombomere, as seen more clearly in the black and white images of SB216763 treated animals included. Cell nuclei labelled with TO-PRO-3. (F) Treatment with 10 µM SB216763 results in 75% increase in fluorescence (P-value ≤  0.001) within rhombomeres, and an increase in fluorescence of 145% at rhombomere boundaries (P-value ≤  0.001). (G) Wild-type embryos were treated with 200 µM ICG-001 and 100 µM Windorphen to inhibit β-catenin mediated transcription. Compared to DMSO controls, treated embryos show reductions in ventricle size. Compared to DMSO controls, treated embryos show little to no reduction in Taz immunofluorescence. Scale bars =  100 µm.

Our data demonstrates that *taz* mRNA is expressed during BVS development. To identify where Taz protein is present, we then utilized a Taz specific immunofluorescence for two purposes: (i) to confirm that *taz* mutants lack detectable protein; and (ii) to confirm our *in situ* hybridization results. In *taz-/-* mutants immunoreactivity for Taz within the hindbrain is largely eliminated, suggesting our mutation is a strong hypomorph or null (Fig 2D). Consistent with our findings from in situ hybridization, Taz protein localizes to a series of stripes within the hindbrain (Fig 2C). Based on morphological landmarks (such as the otic vesicle), these stripes align with rhombomere segment boundaries (Fig 3). This localization agrees with other reports using transgenic animals demonstrating that TEADs, the transcriptional binding partners of Taz, are active at rhombomere boundaries [17].

### Hippo signaling intersects with Wnt signaling in the hindbrain

Canonically, the activity of Taz and Yap is regulated by the activity of the kinases Lats1 and Lats2. Activation of Lats1/2 results in the phosphorylation of Taz and Yap, leading to their degradation or cytoplasmic retention [31]. Current evidence suggests that Taz/Yap can be additionally regulated post-transcriptionally by non-canonical mechanisms. Therefore, we sought to examine signaling pathways known to be active at rhombomere boundaries and what role such pathways may play in post-transcriptional regulation of Taz. Wnt, Notch, RA, and FGF signalling have been shown to regulate segmentation in the hindbrain, with FGF and RA regulating segment-specific gene expression, and Wnt and Notch regulating signalling at segment boundaries. A growing body of evidence suggests that the Wnt pathway interacts with Taz/Yap [35,36]. Given that the hindbrain tissue expresses Hippo and Wnt signaling components, we wanted to test the hypothesis that these two processes might work in concert to regulate hindbrain ventricle morphogenesis. If Taz is regulated by Wnt signalling, there could be multiple ways Taz is regulated: (i) Taz is regulated by β-catenin mediated transcription and is Taz acts downstream of Wnt signaling; or (ii) where Taz and Wnt signaling crosstalk occurs and Taz is post-transcriptionally stabilized by Wnt signaling. To test the hypothesis that Taz may be downstream of β-catenin, we first sought to determine whether Wnt expression was altered within hindbrain of our *taz-/-* mutants (Fig 3A) [42]. Our analysis demonstrated that *taz-/-* mutants robustly express *wnt1* (Fig 3A), but that such domains are highly disorganized.

Since Wnt is positioned appropriately to regulate Taz, we next determined the consequences of inhibiting β-catenin mediated transcription. β-catenin mediated transcription of canonical Wnt transcriptional targets requires the formation of a complex containing β-catenin and T cell family (TCF) members including LEF-1, TCF-7, TCF3b, and TCF4 [43–50] and the binding of coactivators including the histone acetyltransferases CREB-binding protein (CBP) and p300 [51–53]. Knockdown of these coactivators in flies (CBP) and cell culture (p300) results in reduction in Wnt activity [54,55]. In order to block Wnt/β-catenin mediated transcription, we pharmacologically treated embryos with Windorphen and ICG-001 [56–58]. Windorphen has been shown to inhibit the interaction between β-catenin and p300 by disrupting the association of p300 with the C-terminal transactivation domain of β-catenin [56,59]. ICG-001 is a small molecule inhibitor of β-catenin mediated signaling by binding to cyclic AMP response element-binding protein (CBP), thus blocking β-catenin and CBP interaction, and subsequently inhibiting Wnt/β-catenin mediated transcription [60]. We found that treatment of embryos with both β-catenin transcription inhibitors resulted in reduced ventricle size when compared to control embryos (Fig 3B; S5 Fig; 70% reduced, p-value 0.01; M_Wind+ICG_ = 87.47 μm^3^, SD_Wind+ICG_ =  71.48, M_DMSO_ = 148.48 μm^3^, SD_DMSO_ = 23.87).

To complement these studies, we injected a dominant repressive form of *tcf3* mRNA (drTCF3) which lacks the β-catenin interaction domain. Inhibition of B-catenin mediated transcription via drTCF3 similarly reduced ventricle size (S5 Fig C).

Since both Taz and Wnt-mediated signalling is necessary for proper hindbrain ventricle formation, we wanted to test the hypothesis that these two processes might work in concert. Because both Taz and Wnt are active at the rhombomere boundaries, we sought to determine whether Taz was inactivated throughout the hindbrain when Wnt is off. We utilized the tankyrase inhibitor XAV939 to antagonize Wnt activity. XAV939 has been shown to inhibit Tankyrase 1 and 2 in cells, resulting in an increase in Axin-GSK3β complexes and β-catenin destruction activity [61,62]. Treatment of embryos with XAV939 resulted in reduced ventricle size when compared to controls (Fig 3D; S5 Fig; 56% reduced, p-value 0.001; M_XAV939_ = 90.69 μm^3^, SD _XAV939_ =  30.73, M_DMSO_ = 206.95 μm^3^, SD_DMSO_ = 23.05). Strikingly, treatment of embryos with XAV939 also resulted in profound reduction of Taz protein localized at rhomobomere segment boundaries. This supports our hypothesis that in a WNT-off state, Taz protein is degraded within the rhombomere segments. To see the effect of constitutively activating Wnt signaling in embryos, we treated embryos with SB216763, a competitive inhibitor of glycogen synthase kinase 3 [63]. We found that while the ventricles only showed slight reduction in size (40% reduced, p-value 0.001; M_SB216763_ = 99.71 μm^3^, SD_SB216763_ =  34.93, M_DMSO_ = 140.21 μm^3^, SD_DMSO_ = 27.58)., SB216763 treated embryos displayed an increase in detected Taz protein (Fig 3E; S5 Fig). Specifically, there was an increase in signal localized to rhombomere boundaries in addition to increased signal within rhombomeres. Quantification with ImageJ demonstrated that treatment with SB216763 results in a 75% increase in fluorescence within rhombomeres (P-value ≤  0.001) and an increase of 45% at rhombomere boundaries (P-value ≤  0.001, Fig 3F; S5 Fig). While inhibition of Wnt/β-catenin-mediated transcription resulted in a reduced ventricle size, inhibiting β-catenin-mediated transcription did not result in a decrease in Taz immunofluorescence detected at rhombomere boundaries (Fig 3G). This suggests that β-catenin mediated transcription is necessary for proper hindbrain ventricle development but is not required for Taz expression.

This suggests that the hindbrain tissue is a location where Wnt regulates the stability of Taz. Furthermore, constitutive activation of Wnt throughout the hindbrain results in increase of Taz protein detected not just rhombomere boundaries, but within the rhombomeres themselves, where Wnt is typically off.

### Taz is post-transcriptionally regulated by Wnt signalling

**Fig 4:**
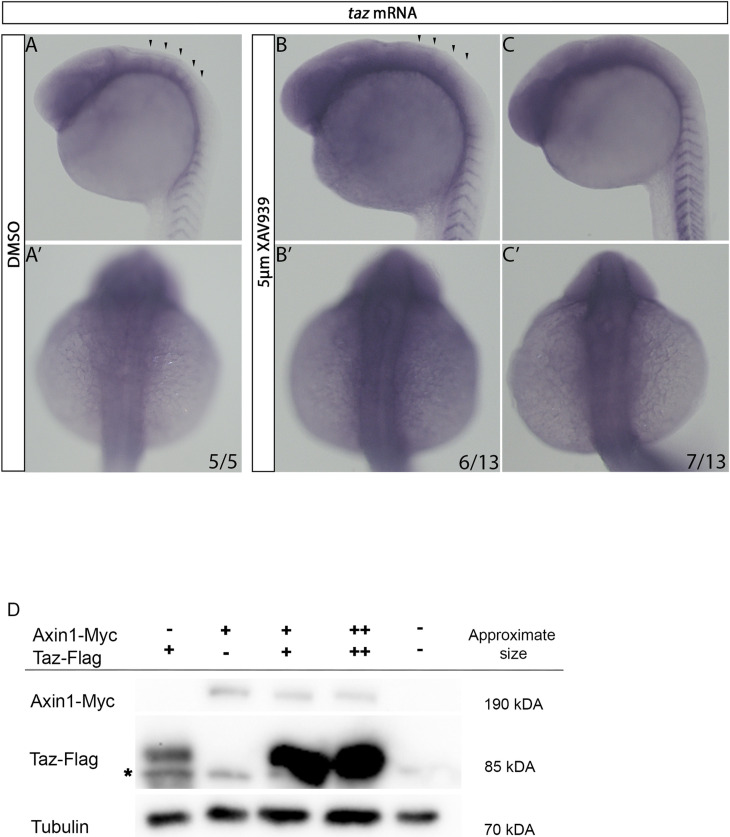
Wnt activity affects *taz* expression at rhombomere boundaries. (A-C’) In DMSO treated animals, at 24 hpf *taz* mRNA is seen broadly in the anterior of the embryo, with visible striations indicative of *taz* mRNA enrichment at rhombomere boundaries shown laterally (A) and dorsally with the hindbrain in focus (A’). In 5 µM XAV939 treated animals, at 24 hpf show a reduction of *taz* mRNA enrichment at the rhombomere boundaries (B-B’) or complete loss of this striated pattern (C-C’). (D) Injection of 200 pg Taz-Flag allowed visualization of the expected band for via western blot (lane 1; note the appearance of a non-specific band labeled *  present in all lanes, including the lane 5 negative control). Similarly, injection of 200pg Axin1-Myc resulted in a band at the expected MW being visualized via 9E10 western blot (lane 2). Co-injection of 200 pg Axin1-Myc and 200 pg Taz-Flag resulted in a greater amount of Flag-Taz detected (lane 3) which was also seen when 400 pg of Axin1-Myc and 400 pg Taz-Flag was injected (lane 4).

In tissue culture, it has been shown that the β-catenin destruction complex is able to sequester and act as a cytoplasmic sink for Taz [35,36]. Conversely, Taz has been shown to act as a downstream element of the Wnt cascade, however, we have shown that Wnt regulates Taz. In the context of the brain ventricle, to investigate the step in which Wnt regulates Taz, we first tested the hypothesis that Wnt affects the amount of *taz* mRNA. To manipulate Wnt signalling, we treated animals with the pharmacological Wnt antagonist XAV939 after gastrulation. In DMSO control animals at 24 hpf, *taz* mRNA expression is seen broadly in the rostral region and an enrichment of *taz* mRNA is seen at the rhombomere boundaries (Fig 4A-A’). Conversely, embryos that were treated with 5 µM XAV939 show a reduction (6/13) or complete loss (7/13) of *taz* mRNA enrichment at the boundaries (Fig 4. B-C’). Interestingly, Interestingly, in XAV939 treated animals, *taz* mRNA is overtly normal in both rostral and somitic regions, suggesting that Wnt functions to regulate Taz specifically in the hindbrain.

We then focused on the possibility of crosstalk between the β-catenin destruction complex and Taz. As Axin is a scaffolding protein suggested to be the rate limiting factor for β-catenin destruction complex formation, and increased Axin stability has been shown to result in a subsequent increase in β-catenin destruction complexes formed, we chose to assay the relationship between Taz and Axin1 [62,64]. We sought to overexpress *axin1* to determine if we detect any changes to Taz protein level. To overcome the lack of specific antibodies towards either Axin1 and Taz in zebrafish, we placed a Myc epitope tag (Myc) on the C-terminus of Axin1 and a Flag-tag on the C-terminus of zebrafish Taz. Injection of mRNA encoding Axin1-Myc or Taz-Flag individually (with respective tag controls) showed bands at the predicted molecular weight (Fig 3D, lane 2 and 3). Co-injection of Taz-Flag and Axin1-Myc resulted in an increase in detected Taz-Flag protein (Fig 3D, lane 4). This suggests that the previously identified interaction between the murine β-catenin destruction complex and Taz is likely conserved in zebrafish.

Our results demonstrate that Wnt regulates Taz post-transcriptionally. Localization of *taz* mRNA at rhombomere boundaries is dependent on Wnt activity as loss of Wnt signalling results in loss of *taz* mRNA detected. Additionally, Taz protein stability at rhombomere boundaries is dependent on Wnt activity as shown previously by XAV939 and Taz immunofluorescence which is additionally supported by tagged *taz* and *axin1* mRNA overexpression and Western blot experiments.

### *taz-/-* mutants show disorganization of apicobasal polarity components and changes to gene expression of boundary specific genes

In previous studies, complete or partial failure of brain ventricle expansion has been linked to reduced cellular proliferation, but not to changes in the number of cells undergoing apoptosis [1]. Similarly, we wanted to investigate cellular proliferation in *taz-/-* mutants. We saw that the *taz-/-* mutants had only a slight reduction in the number of proliferating cells and no changes in the number of apoptotic cells when compared to wild-type animals (S6 Fig A-B). This suggests that the nature of the brain ventricle phenotype in *taz-/-* mutants is similar to other early brain ventricle formation mutants, and Taz is necessary for specifying apicobasal cell polarity in hindbrain ventricle formation [37].

**Fig 5:**
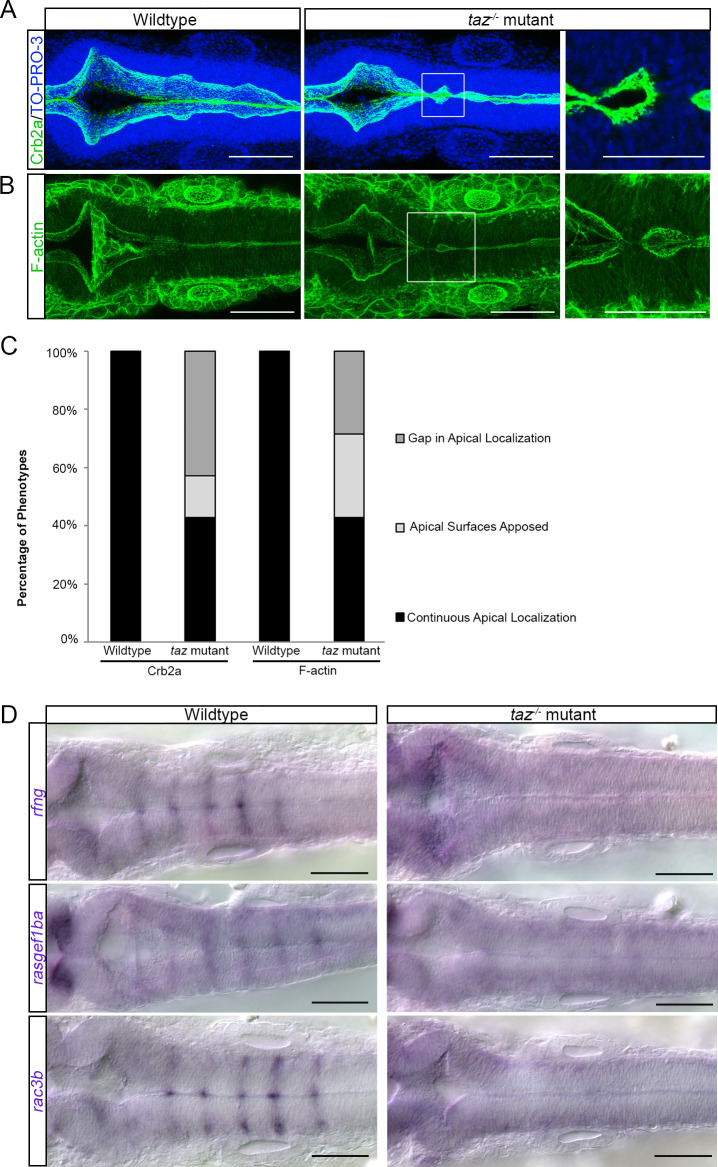
Apicobasal polarity components and patterned gene expression is perturbed in *taz*^-/-^ mutants. Changes to apicobasal polarity and cytoskeletal organization were assayed in wild-type and *taz-/-* mutants. (A) Crb2a localization in wild-type animals outlines the apical side of the ventricle. In *taz-/-* mutants, this localization is disturbed at locations where the ventricle fails to separate. Cell nuclei labelled with TO-PRO-3. (B) Phalloidin staining of F-actin in wild-type animals is seen strongly at the apical side of the ventricle lips, whereas in *taz-/-* mutants, locations where failure of midline separation occurs, we observe disturbances to F-actin organization. (C) Quantification of embryos observed having continuous, apposed, or gaps in apical localization. In wild-type animals, apical localization of Crb2a and F-actin is seen continuously from the anterior to posterior axis of the hindbrain ventricle, whereas in *taz-/-* mutants, a large proportion show either a gap or apposition of apical surfaces. (D) In *taz-/-* mutants, expression of genes that appear in a striated pattern are lost. These genes include *rfng*, *rasgef1ba,* and *rac3b.*

In development, tissues undergo drastic morphogenesis events to create the final organ shape [65]. In particular, in tissues where two opposing sections must migrate and separate, the failure of this separation can be attributed to poor integrity of the epithelia and/or the absence of junctional proteins [1,66]. Therefore, we sought to investigate whether failure of hindbrain ventricle lips separation is due to disorganization in epithelial and junctional proteins. In zebrafish, mutations in *pals1a*, (*protein associated with LIN7 1*), *prcki* (*protein kinase C, iota*) and *crb2a (crumbs cell polarity complex component 2a)* display midline separation defects in brain ventricle morphogenesis due to the disruption of apicobasal polarity [37,67–71]. Crb2a is known to localize in a complex with other apicobasal polarity proteins on the apical side of the neural epithelium [72]. Therefore, we used Crb2a as an indicator of apicobasal organization and neuroepithelium integrity in our *taz-/-* mutants. In wild-type embryos, we found that Crb2a protein is localized on the apical side of the hindbrain ventricle (Fig 5A). In *taz-/-* mutants, we found that 57% had disorganized Crb2a localization in the hindbrain ventricle. Specifically, 43% of *taz-/-* mutants display a discontinuity of Crb2a localization (Fig 5A, C), and 14% of *taz-/-* mutants show aberrantly apposed apical surfaces (Fig 5A, C WT 0/10, *taz-/-* mutants: 6/14 gaps, 2/14 apposed surfaces, Fischer’s exact P < 0.001).

Given our observation of apicobasal disruption, we next assayed F-actin to visualize whether there was cytoskeletal disorganization in *taz-/-* mutants. In wild-type embryos, F-actin is localized to the apical side of the hindbrain ventricle (Fig 5B). Comparatively, in 57% of *taz-/-* mutants, F-actin localization is disrupted. Specifically, 28.6% of *taz-/-* mutants display a gap in apical localization, and a further 28.6% of *taz-/-* mutants show aberrantly apposed apical surfaces (Fig 5B, C, WT: 0/8, *taz-/-* mutants: 2/7 gaps, *taz-/-* mutants: 2/7 apposed, Fischer’s exact P < 0.01).

Developmental structural changes are a consequence of changes to gene expression. As Taz is a co-transcriptional regulator and is localized to the boundaries, we looked at known markers of boundaries to see if their expression is perturbed. In wild-type animals, *radical fringe* (*rfng*), *RasGEF domain family, member 1Ba* (*rasgef1ba*) and *Rac family small GTPase 3b* (*rac3b*) are expressed at near rhombomere boundaries, which is supported by other published data ([73–75]; Fig 5D). In *taz-/-* mutants, this expression pattern for *rfng, rasgef1ba,* and *rac3b* are lost (Fig 5D).

**Fig 6:**
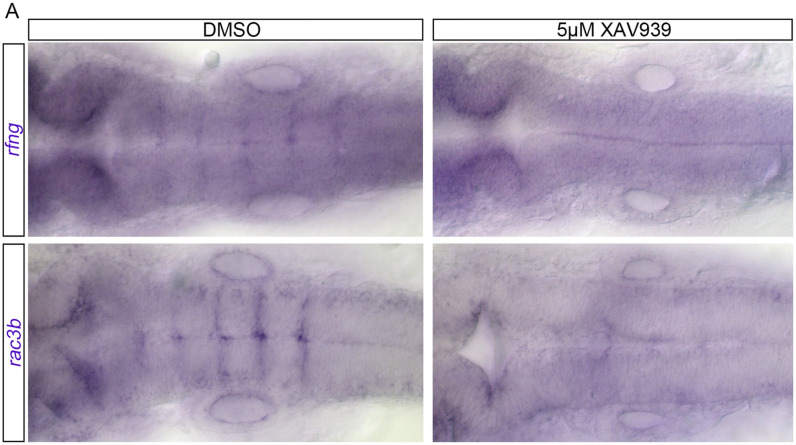
Wnt activity affects boundary cell gene expression. In DMSO treated animals at 24 hpf *rfng* mRNA is expressed at rhombomere boundaries (15/15). In 5 μM XAV939 treated animals at 24 hpf this striated expression pattern is lost (23/23). In DMSO treated animals at 24hpf *rac3b* is also expressed at boundaries (16/16). In 5 μM XAV939 treated animals at 24 hpf this striated expression pattern is lost (23/23).

Additionally, to further address our hypothesis that Wnt may affect Taz stability, we performed a Wnt antagonist experiment with XAV939. In Taz mutants we see that the rhombomere boundary genes *rfng* and *rac3b* are reduced. If the β-catenin destruction complex negatively regulates Taz stability, we predict that treatment with XAV939 should result in a reduction of rhombomere boundary gene expression. In DMSO treated animals at 24 hpf, we see *rfng* and *rac3b* expressed in a striated pattern at the rhombomere boundaries (Fig 6). However, in 5 μM XAV939 treated animals at 24 hpf this striated expression pattern is lost for both *rfng* and *rac3b*.

Our results demonstrate that while there are little to no changes to cell proliferation and cell death in *taz-/-* mutants, there are changes to the cytoskeletal architecture and apicobasal polarity. Additionally, expression of boundary specific genes are perturbed.

## Discussion

Although prior research implicated Hippo signalling in vertebrate hindbrain development [17,33], the role of Hippo signalling within BVS development remained unclear. Here, we have described a clear role for the Hippo-regulated transcription factor Taz in hindbrain ventricle morphogenesis as mutants that lack Taz protein have reduced hindbrain ventricle sizes. We also show that this reduction of size in the hindbrain ventricle is due to a failure of the ventricle lips from separating. Furthermore, we show that the hindbrain ventricle formation is an *in vivo* developmental process that requires crosstalk between the Wnt and Hippo signalling pathways, specifically Taz. We demonstrate that within the hindbrain, that Taz is post-transcriptionally regulated by Wnt signalling through pharmacological manipulation. Additionally, we show that *taz-/-* mutants have disorganized apicobasal and cytoskeletal components and changes to gene expression *rfng, rasgef1ba* and *rac3b*.

In this study, we show that loss of Taz results in disruption of midline separation during hindbrain ventricle formation. To generate embryos that lacked functional Taz protein, we utilized TALENS that disrupted the coding region of exon 1. This targeting site sits prior to the conserved TEAD, WW, and PDZ binding domains. Lack of additional internal methionines 3’ to the frameshift suggests the lack of functional protein in such strains. In addition, any truncated Taz amino acid products would contain the epitope recognized by the Taz/Yap antibody, and these protein products are not recognized by the immunofluorescence experiments in Taz mutants. Furthermore, our *taz-/-* mutants behave similarly to other *taz-/-* mutants published in literature. Adult female *taz-/-* mutants are infertile – they produce eggs that cannot go undergo cleavage [76]. However, *taz-/-* mutant males are capable of fertilizing eggs produced from *taz*^* + /-*^ heterozygote or wild-type females [76] Click or tap here to enter text.. Finally, we confirmed the specificity of the *taz* mutation using *taz* mRNA rescue experiments. Using full length and various truncated forms of Taz protein, we found that only the full length and the truncated Taz protein containing the TEAD binding domain were able to rescue the hindbrain ventricle phenotype in Taz mutants, suggesting that lack of TEAD binding activity and subsequent loss of Taz-mediated TEAD transcription results in brain ventricle phenotypes.

Previous work studying zebrafish brain ventricle morphogenesis has categorized mutants into categories based on the phenotypes present as well as the stage in which BVS morphogenesis is altered. These categories include the first initial brain ventricle development phase from 17 hpf to 24 hpf, and the second phase, brain ventricle expansion from 24 hpf to 26 hpf. Within the initial brain ventricle development stage, four subcategories are within. These four subcategories of the “initial brain shaping and inflation mutants” include: 1) midline separation defects (where the ventricle or rhombic lips fail to separate from each other during brain ventricle development); 2) reduced ventricle size (where ventricle midlines separate but do not fully inflate and are reduced in volume/size); 3) midbrain-hindbrain boundary (MHB) defects (where the brain ventricles are also reduced in size, but the key characteristic is that the MHB appears abnormal in shape); and 4) absence of lumen inflation (where mutants appear to not have ventricles, and rhombic lips appear to remain sealed together from anterior to posterior). Mutants in these four classifications have a phenotype onset of 18-21 hpf, whereas later brain ventricle mutants appear normal until 28 hpf. Thus, we sought to determine where Taz would be necessary within the current model of brain ventricle development.

### Hindbrain ventricle morphogenesis requires Hippo and Wnt Crosstalk

Previous reports have demonstrated the role of canonical Wnt within hindbrain development – in *Xenopus laevis*, loss of Wnt has been attributed to a decrease in Hox gene expression; in mice, Wnt knockout results in a loss of midbrain and anterior hindbrain cell fate [77,78]. The zebrafish model has been useful in demonstrating the role of Wnt in the hindbrain, where a series of Wnt factors (that often overlap and act together) dictate the development of the hindbrain – for example, Wnt1 and Wnt10b are necessary for gene expression within the midbrain hindbrain boundary (MHB), and in addition to this, Wnt3a has been shown to be necessary when there is a loss of Wnt3a [19,79].

Prior research has implicated an interaction between Wnt and Hippo crosstalk, but the evidence for this during embryonic development has so far been elusive. There are several tissues where Wnt and Hippo are both active. For example, Wnt is necessary for development of the eye, RPE, heart and neural crest [80–82]. Hippo signaling is similarly involved in the development of each of these components, yet no interactions have been noted [83–85]. *wnt1* mRNA is expressed at rhombomere boundaries, and we note that Taz protein is stabilized in such cells. We know that Taz-TEAD complexes are nuclearly localized and active in such cells [33], but it is plausible that such activation and stabilization are simply the result of repression of Taz phosphorylation. We provide a strong argument in favor of a more complex scenario. Using Wnt pathway antagonists, we have demonstrated that loss of Wnt activity results in a reduction in stable Taz protein. Furthermore, ectopic activation of Wnt results in a global increase in Taz protein throughout the hindbrain. In comparison, when pharmacologically manipulating β-catenin mediated transcription no change to Taz protein abundance was observed. Therefore, we propose a model in which Taz is regulated by upstream components of Wnt signalling, namely Axin1 and the β-catenin destruction complex, rather than β-catenin mediated transcription. While we observed the stabilization of Taz protein when Axin1 was overexpressed, this somewhat contradicts our XAV939 experiments, which show a reduction of both Wnt and Taz. This could represent differing biological activities of Axin1 and Axin2 (the target of XAV939), or an ability of overexpressed Axin protein to function separately from the destruction complex.

### The role of Taz in apicobasal polarity and cytoskeletal organization

In our study we found that *taz-/-* mutants show midline separation defects consistently at 22 hpf. This phenotype resembles initial brain shaping mutants such as *crumbs2a* (*crb2a)*, *protein kinase c iota* (*prkci)*, and *protein associated with lin7 1* (*pals1a*, and previously known as *mpp5a)*, which are all mutations in genes that are known to affect apicobasal polarity. Our analysis of *taz-/-* mutants has clearly demonstrated that apicobasal polarity is altered within the developing hindbrain. Immunofluorescence analysis of Crb2a and F-actin localization demonstrated loss of polarity, which was typically concentrated in positions where the brain ventricle remained attached.

Mechanical signals regulate Taz/Yap outside of Hippo signalling, suggesting that there are mechanisms that regulate Taz outside of the core Hippo kinase cascade (reviewed in Varelas, 2014) [86]. Morphogenesis undergoes and requires a series of changing cell-cell contacts, which then affects cell proliferation and migration [87]. These establishing cell contacts inhibit Taz/Yap activity by phosphorylation and sequestration of these proteins in the cytoplasm (reviewed in Varelas, 2014). Additionally, in vitro analyses demonstrate TAZ and YAP bind to the Crumbs polarity complex [88].Thus, dynamic cell contacts, mature junctions and assembly of the Crumbs complex lead to TAZ/Yap inactivation. However, our work posits that this relationship may be reciprocal, as *taz-/-* mutants have disrupted apicobasal polarity as seen by changes to the localization of the Crumbs complex and that Taz is necessary for proper apicobasal polarity in the hindbrain. This is also seen in Yi et al., 2019 [89], the micropyle is enriched with Taz protein, a structure with dynamic levels of actin and tubulin, and they propose that co-localization of these components suggest that Taz has a role in cytoskeletal organization. In the micropylar cell, Taz accumulation and cell fate specification leads to changes in actomyosin, microtubule, and intermediate filament cytoskeletal components, thus facilitating changes to cell shape and size [90]. In other developmental structures, the cytoskeleton and apicobasal polarity components interact or are regulated by Taz. We also demonstrate that in the hindbrain, Taz is necessary in either initiating hindbrain apicobasal polarity or maintaining polarity during hindbrain morphogenesis.

We demonstrate that Taz protein is localized to rhombomere boundaries in the zebrafish hindbrain, similar to the spatial localization reported for TEAD-dependent transgenic reporter strains [17,33]. In the zebrafish hindbrain, Taz localization has been linked to Ephrin-mediated actomyosin phosphorylation, and the subsequent contraction of tissue at segment borders [33]. In congruence with Cayuso et al., 2019, the Pujades group demonstrate that within the zebrafish hindbrain, boundary cells act as hubs of mechanotransduction signals, as they are sensitive to mechanical stress, and that this mechanotransductive signal is mediated by Yap/Taz-TEAD activity [17]. Specifically, Yap/Taz-Tead activity is necessary for the proliferative state of progenitors. Although we have not observed an alteration of proliferation in *taz-/-* single mutants, Voltes et al., 2019 posit a redundancy with Yap/Taz in this regard, which may explain the discrepancy.

In our study, we demonstrate that *taz-/-* mutants show a loss of defined *rfng* expression, a gene that is expressed only in hindbrain boundary cells. Our results accord with those of Cayuso et al., 2019 in which they observe a decrease in *rfng* expression in Taz morpholino knockdown and in Taz mutants. While *rfng* is a marker of boundary cells, it also has a role in Notch mediated signaling and this has been demonstrated in the zebrafish hindbrain [27]. Significantly, knockdown of *rfng* has been shown to disrupt *wnt1* expression as well as result in a reduced ventricle size [27,91]. Thus, the exploration of Notch and its role in boundary cell proliferation and identity is an avenue that necessitates future exploration. Furthermore, in *taz-/-* mutants, expression of *rac3b* and *rasgef1ba* are also decreased. Literature has shown that boundary cells that express *rfng* also express *rac3b* and *rasgef1ba* [92]. Furthermore, *rac3b* has been shown to lie in the genome close to *rfng* [93]. Rac3b is a small-GTPase that affects Eph/Ephrin signalling and downstream actinomyosin assembly [93]. Additionally, Rasgef1ba is a guanine nucleotide exchange factor (GEF) [73]. Given that boundaries and cell segregation in the hindbrain are partially dependent on physical mechanisms such as actomyosin cables, much work has proposed that GTP regulatory elements are the primary mechanism by which these physical boundaries are regulated [94]. Our work posits that Taz may be upstream of such regulatory elements. However, work done by the Pujades group has shown that knockdown or downregulation of Rac3b results in decreased Taz/Yap/TEAD activity, suggesting that mechanical cues are both regulated by and responded to by Taz/Yap/TEAD [17]. Since several boundary specific genes are changed in Taz mutants, our work necessitates the further study of boundary cells in the context of Taz. If Taz regulates boundary cell identity, other boundary specific genes must be explored. Consequently, if the loss of Taz results in a phenotypically aberrant hindbrain ventricle, exploring if the loss of genes such as *rfng*, *rac3b* and *rasgef1ba* phenocopies the *taz-/-* mutant, thus providing a mechanism for our phenotype.

## Materials and methods

### Zebrafish care and line maintenance

Animals were housed and treated according to protocols approved by the Animal Care and Use Committee of the University of Alberta (AUP 00000082). Zebrafish embryos were obtained by breeding adult zebrafish using standard methodology. Embryos were grown to appropriate stages at 25.5℃–33℃ in accordance to [95]. The *taz* mutation was maintained as a stable heterozygote line (*taz*^* + /-*^), and in-crossed to obtain *taz-/-* mutants. The *yap*^* + /-*^ mutation was maintained as a stable heterozygote line, and in-crossed to obtain *yap-/-* mutants. Genotyping was performed using PCR product size. The sequence for genotyping primers are as follows: *taz* FWD 5’- CCA TCG GCC ATT TTA ATC GAA G-3’, REV 5’- AAA GAG CCT CCA GAT CCG TGT C-3’, *yap* FWD 5’- CAG TTT CTC CTG GTG CAC TGA -3’, REV 5’ – GGC ATG TCA TCA GGT ATC TCG T – 3’. Unless otherwise stated, AB zebrafish strains were used.

### Whole mount *in situ* hybridization

*In situ* hybridization was performed as described previously [96]. Probe synthesis for *in situ* hybridization were as follows: probes were labeled with antisense digoxigenin-UTP with T3, T7 or SP6 polymerase (Sigma) and were subsequently purified using SigmaSpin Post-Reaction Clean-Up columns.

Probes included both plasmid based: *atoh1a, her6, ascl1a, ascl1b, neurod4, neurod1, sox1b, sox2, sox3, insm1a, ngn1* as well as PCR primer based, *taz* FWD 5’- CCT CCA TAA TCA GGT CTC CAA C -3’, REV 5’- ATT TAG GTG ACA CTA TAG GCA CAA ATC CGA CAG CTA AAG-3’, *wnt1* FWD 5’-GCC ATT ACA AGT GCT GGT GTT A-3’, REV 5’ -TAA TAC GAC TCA CTA TAG GGC TGA AGC ATG CGT TTC AGA TAG -3’, *rfng* FWD 5’-ACT ATG TGA TCC TGC CCA GTC T-3’, REV 5’- TAA TAC GAC TCA CTA TAG GGA GCC ACC CTG TTT CTT CAT TTA-3’.

### Mutant generation/ transcription activator-like effector nuclease (TALEN) construction

Transcription activator-like effector nucleases (TALENs) were used to generate *taz* homozygous mutants (*taz*^ua1015^). This protocol was based on [97]. TALEN target sites were selected, and primers were designed using Mojo Hand 2 [98]. Each TAL construct was then assembled using the FusX system. TAL construct mRNA was synthesized using the SP6 mMessage mMachine kit, and mRNA injections were performed at the one cell stage using 200-400 pg of either TAL mRNA. The *taz* TALEN target site: TGA-GCG-GTA-ATC-CTC-TCC-AGC-CGA-TAC-CGG-GCC-ACC-AGG-TGA-TCC-ATG-TCG-CCA. To generate *yap* mutants, CRISPR-Cas9 mutagenesis was utilized. The protocol was based on [99]. The *yap* target site: GAC-TGG-CGG-AGG-TGC-TGA-GG**T-GG.**

### Dextran injections

Hindbrain injections with 10 mg/mL Texas Red Dextran (10 000 MW Life Technologies) in Danieau Buffer were performed as according to [39]. After anesthesia with tricaine methanesulfonate, appropriately staged embryos were embedded into agar, and Texas Red Dextran was injected into the hindbrain.

### Immunohistochemistry

Standard protocols for immunohistochemistry data were used. Briefly, embryos were collected at appropriate stages, fixed in 4% PFA, permeabilized and were subsequently blocked in an appropriate solutions. If necessary, antigen retrieval via 10 mM Citric Acid was used. Additionally, TO-PRO3 nuclear staining at a 1:1000 dilution was used when appropriate. Antibodies for Crb2a Zs4 (ZIRC), F-actin (Alexa Fluor 488 Phalloidin Invitrogen), Phospho-Histone-H3 (BD Bioscience), Active Caspase 3, Yap (Cell signaling 4912) and Yap/Taz (Cell signaling 8418) were used between 1:200 to 1:1000 dilution, subsequent Alexa Fluor Dyes secondary antibodies were used from 1:800 to 1:1000 dilution.

### Measuring Fluorescence Intensity

For Taz immunohistochemistry, 4-5 regions at rhombomere or rhombomere boundaries were selected, and density and area were measured using ImageJ. Background mean grey value was measured in 4 adjacent regions. Measurements were averaged for each image, and subsequently used to determine averages for treatments. Corrected fluorescence was calculated, and analyzed using Analyses of Variance (ANOVA) followed by post-hoc Tukey’s Honest Significant Difference Test.

### Pharmacological treatments/ signaling inhibition

To inhibit β-catenin mediated transcription, *drTCF3* mRNA, a dominant repressive form of Tcf3 was injected into embryos, courtesy of (courtesy of Richard Dorsky, University of Utah). All compounds were dissolved in dimethyl sulfoxide (DMSO) and diluted in embryo media to achieve working concentrations. Embryos were treated with 200 µM of ICG-001 and 100 µM Windorphen to inhibit Wnt signaling. 5 µM solution of XAV939 (Sigma) was used as an antagonist to Wnt activity and applied to embryos at tailbud (10 hpf). 10 µM solution of SB216763 (Sigma) was used as an agonist to Wnt activity and applied at tailbud (10 hpf) or 2hpf.

### Cloning and mRNA synthesis

Total mRNA was extracted using the RNAqueous-4PCR Total RNA Isolation kit (Invitrogen/Ambion). cDNA was then isolated using the SuperScript III One-Step RT-PCR System with Platinum Taq DNA Polymerase (Invitrogen) and cloned into the appropriate vector system. For Western Blot experiments, *taz* and *axin1* mRNA were cloned into pT7TS-T7 and pCS3-MT vectors to synthesize Flag-Tagged Taz and Myc-Tagged Axin1. Taz Primer sequence: FWD: 5’ – TAT AAG ATC TCC ATG AGC GGT AAT CCT CTC CAG CC – 3’ REV: 5’ – TAT AAC TAG TCT AGA GCC AGG TGA GGA AGG GCT CG – 3’ Axin1 Primer sequence: FWD: 5’ – TAT AAG ATC TCT ATG AGC ATG AGT GTA AAC GAG AAG G – 3’ REV: 5’ – TAT ACT CGA GTC AGT CGA CCT TCT CCA CTT TTC CG – 3’

Injected mRNA was synthesized by linearizing plasmid DNA and use of SP6 or T7 mMessage mMachine Kits (Invitrogen/Thermo Fisher Scientific). RNA recovery and cleanup was done with Amicon Ultra Centrifugal Filters (Millipore).

### mRNA injection

Synthesized mRNA was diluted to working concentrations in DEPC treated water (0.1% diethyl pyrocarbonate) and injected into 1-cell stage embryos. Concentration of mRNA injected was calculated using bolus size on a micrometer. Injections were performed on ASI MPPI-2 Pressure Injector (Applied Scientific Instruments).

For *eGFP*, *drTCF3*, and modified Taz construct injection, after injection into 1-cell stage embryos, embryos were grown to appropriate stages and analyzed for ventricle phenotypes. In order to account for differences in total mRNA *taz-Flag* and *axin1-*Myc were co-injected with control mRNA that does not interact with Hippo or Wnt signalling. For western blots experimental categories were as follows: 200 pg of *taz-Flag* mRNA with a 200 pg non-reactive *Flag* control; 200 pg of *axin1-Myc* with a 200 pg non-reactive *Myc* control; coinjection of 200 pg of *taz-Flag* mRNA and 200 pg of *axin1-Myc*. After injection, embryos were grown to appropriate stages and harvested for protein.

### Western blot

Embryos were staged and protein was harvested at tailbud (10 hpf) or 70% epiboly (8 hpf). Embryos were dechorionated with a combination of forceps and Pronase E. Embryos were then deyolked and protein was harvested according to [100]. Western Blot analyses were performed on 15-25 embryos per sample. Using “verified” constructs with a Myc or Flag tagged protein, inert and does not react with Taz or Axin1 as control.

### Image preparation and figure generation

Whole embryo images were taken on Olympus SZX12 stereomicroscope (Olympus) with a Micropublisher 5.0 RTC camera and QCapture Suite PLUS Software v3.3.1.10 (QImaging). Confocal images for whole mount *in situ* hybridization and immunofluorescent experiments were taken on Zeiss AxioImager Z1 compound scope using and AxioCam HR camera and Axiovision SE64 Rel.4.8 software (Zeiss) or a Zeiss LSM 510 confocal microscope using Zeiss Zen software. Figures were assembled and produced in Adobe Photoshop and Adobe Illustrator

### Statistics

Several statistical analyses were performed. To analyze size differences of ventricles between wild-type and *taz-/-* mutants, unpaired, two-tailed T-tests were used. To analyze changes to Crb2a localization and F-actin organization between wild-type and *taz-/-* mutants, Fisher’s exact tests were used. *P* values less than 0.05 were considered significant and are differentiated by asterisks when denoted in figures. * *P* < 0.05, **P < 0.01, ***P < 0.001

## Supporting information

S1 Fig *taz*-/- mutant ventricle phenotype is rescued when injected with PDZ containing Taz mRNA.
(A) Embryos were injected with the indicated mRNA at the single cell stage and were fixed at 24hpf. Ventricles were visualized using atoh1a ventricle size of embryos within each group were measured, averaged, and compared amongst each other via ANOVA analysis. A one-way ANOVA revealed that there was a statistically significant difference the ventricle size between three treatment groups (F(3, 29) = [22.05], p < 0.00001). Tukey’s HSD Test for multiple comparisons found that the mean value of ventricle sizes was significantly different between *taz*^+/+^ wild-type + 125 pg eGFP mRNA (M= 241.01 µm3 SD= 36.67) versus *taz^-/-^* mutant + 125 pg eGFP mRNA (M= 146.50 µm3 SD= 21.00 p = 0.00001). Tukey’s HSD Test for multiple comparisons found that the mean value of ventricle sizes was significantly different between taz-/- mutant + eGFP mRNA (M= 146.50 µm3 SD= 21.00) versus taz-/- mutant + 100pg taz+/+ wild-type mRNA (M =188.02 µm3 SD = 41.26, p = 0.031). (B) Gene diagrams of Taz mRNA constructs, including full length *taz*, and various truncated *taz* mRNAs. (C) Embryos were injected with the indicated mRNA at the single cell stage and were fixed at 24hpf. Ventricles were visualized using atoh1a ventricle size of embryos within each group were measured, averaged, and compared amongst each other via ANOVA analysis. A one-way ANOVA revealed that there was a statistically significant difference the ventricle size between five treatment groups (F(4, 55) = [33.33], p < 0.00001). Tukey’s HSD Test for multiple comparisons found that the mean value of ventricle sizes was significantly different between *taz*^-/-^ mutants injected with eGFP (M = 146.50 µm3 SD = 21.00) and Taz ΔWW (M = 75.14 µm3 SD = 30.29 p = 0.00005), e GFP and ΔPDZ (M = 217.77 µm3 SD = 37.96 p = 0.00005), but no significant differences between eGFP injected compared to Taz ΔCC (M 120.33 µm3 SD 33.55 p = 0.36) or Taz ΔTA (M 141.20 µm3 SD 39.81 p = 1.00). Representative images of embryos with ventricle visualized with *atoh1a* in situ of embryos at 24 hpf. Scale bars = 100 µM.2023.
(TIF)

S2 Fig Midline separation defects in *taz-/-* mutants result in reduced ventricle size.
Quantification of brain ventricle size differences between wild-type (grey) and *taz-/-* mutants (black) after *atoh1a in situ*. At all stages examined (22-36 hpf) *taz-/-* mutants showed reduced ventricle size. 22 hpf: *taz*^* + / +*^ wild-type M = 185.46 µM, SD = 37.58 µM versus *taz-/-* mutant M = 99.33 µM SD = 29.09 µM; t(19) = 5.83, p < 0.0001.; 24 hpf: *taz*^* + / +*^ wild-type M = 206.85 µM; SD = 27.97 µM versus *taz-/-* mutant M = 101.60 µM; SD = 32.45 µM; t(21) = 8.29, p < 0.0001.; 28 hpf: *taz*^*+ / +*^ wild-type M = 225.92 µM; SD = 37.00 µM versus taz mutant M = 102.01 µM; SD = 36.69 µM; t(15) = 6.63, p < 0.0001.; 36 hpf: *taz*^* + / +*^ wild-type M = 279.69 µM; SD = 11.82 µM versus *taz-/-* mutant M = 157.77 µM; SD = 48.88 µM; t(19) = 5.95, p < 0.001.
(TIF)

S3 Fig *yap-/-* mutants do not show ventricle phenotypes
A) *atoh1a in situ*s were performed on wildtype and *yap*^*‑/-*^ mutants. At 22 hpf and 24 hpf, *yap-/-* mutant ventricles are comparable to wild-type. B) Taz/Yap immunofluorescence was performed on wildtype and *yap-/-* mutants. In wild-type embryos, signal is picked up within cells at the rhombomere boundaries. In *yap-/-* mutants, this signal at the boundaries is not diminished. Cell nuclei labelled with TO-PRO-3. Scale bars =  100 µM.
(TIF)

S4 Fig *taz* expression is enriched before and during BVS development and is decreased at later stages.
(A) *in situ* hybridization analyses show that *taz* mRNA is expressed broadly at 16 hpf with enrichment in neural tissues, shown laterally (A) and dorsally (A’, A”). (B-B”) At 18 hpf, the start of ventricle shaping, *taz* maintains broad expression throughout the embryo, however, at 20 hpf (C-C”) *taz* is enriched at rhombomere boundaries, and this enrichment persists though 22 hpf (D-D”) and to 24 hpf (E-E”).
(TIF)

S5 Fig Wnt affects hindbrain ventricle size.
Quantification of brain ventricle size differences between DMSO controls and pharmacological manipulation of components of the Wnt pathway. XAV939 treated animals had significantly reduced ventricles (56% reduced, p-value 0.001) when compared to DMSO controls (M_XAV939_ = 90.69 μm^3^, SD _XAV939_ =  30.73, M_DMSO_ = 206.95 μm^3^, SD_DMSO_ = 23.05). Windorphen and ICG-001 treated animals had significantly reduced ventricles (70% reduced, p-value 0.01) when compared to DMSO controls (M_Wind+ICG_ = 87.47 μm^3^, SD _Wind+ICG_ =  71.48, M_DMSO_ = 148.48 μm^3^, SD_DMSO_ = 23.87). SB216763 treated animals had significantly reduced ventricles (40% reduced, p-value 0.001) when compared to DMSO controls (M_SB_ = 99.71 μm^3^, SD _SB_ =  34.93, M_DMSO_ = 140.21 μm^3^, SD_DMSO_ = 27.58).
(TIF)

S6 Fig *taz^-/-^* mutants show no change in proliferation or cell death
A) To assay cell proliferation, phospho-histone-H3 immunohistochemistry was performed to label actively proliferating cells. Compared to wild-type animals, *taz-/-* mutants show mild changes to proliferation. Cell nuclei labelled with TO-PRO-3. B) To assay cell death, cells were labelled using anti-active Caspase-3 (red). Compared to wild-type animals, *taz-/-* mutants show little to no changes in cell death. *Tg(lbx1b:eGFP)* was used to visualize the neural tube, cell nuclei labelled with TO-PRO-3.
(TIF)
