# Supplementary figures and images for: Functional role for Taz during hindbrain ventricle morphogenesis
Source: PLoS One. 2025 Mar 13;20(3):e0313262. doi: 10.1371/journal.pone.0313262 (PMC11906067; doi:10.1371/journal.pone.0313262)

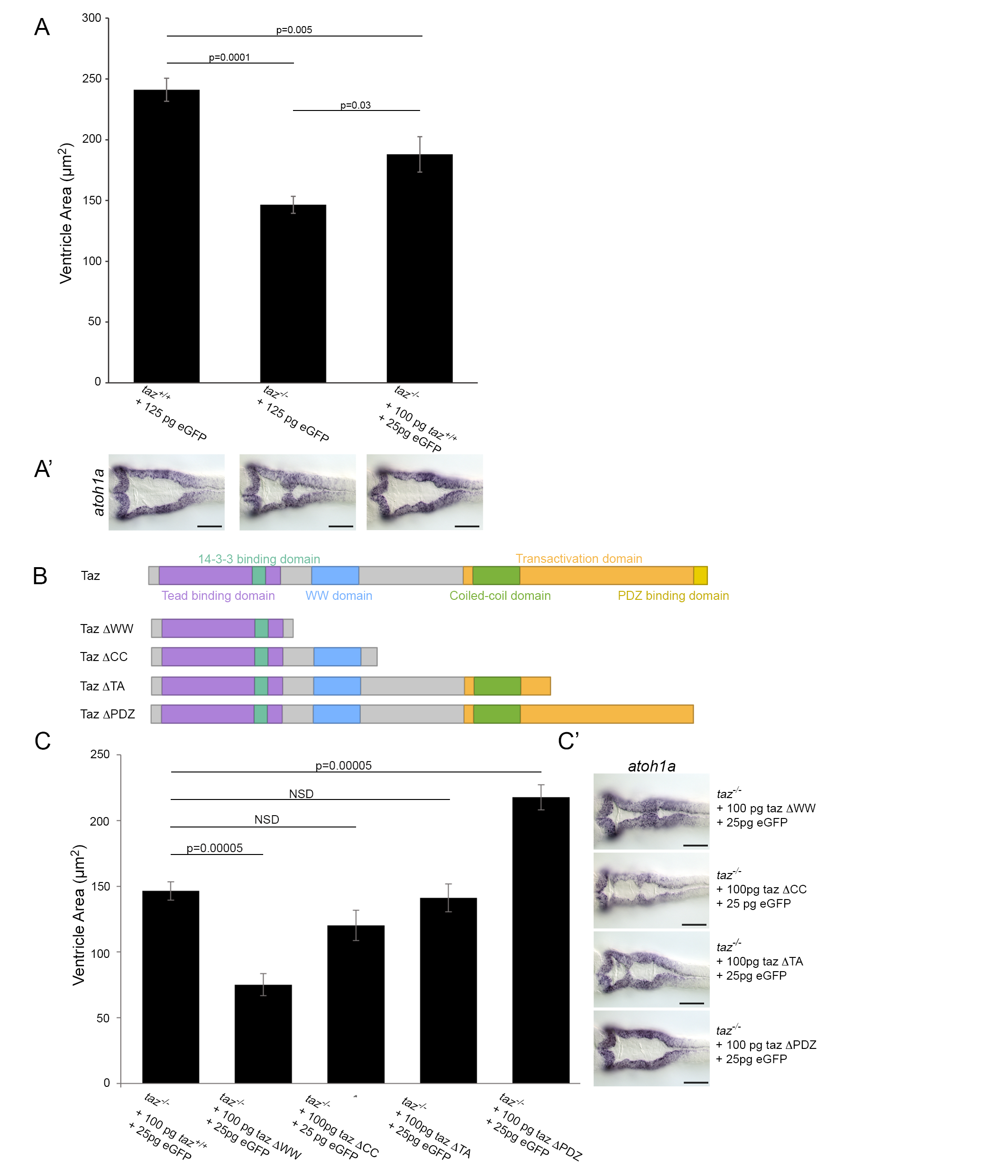

Supplement: S1 Fig — (A) Embryos were injected with the indicated mRNA at the single cell stage and were fixed at 24hpf. Ventricles were visualized using atoh1a ventricle size of embryos within each group were measured, averaged, and compared amongst each other via ANOVA analysis. A one-way ANOVA revealed that there was a statistically significant difference the ventricle size between three treatment groups (F(3, 29) = [22.05], p < 0.00001). Tukey’s HSD Test for multiple comparisons found that the mean value of ventricle sizes was significantly different between taz+/+ wild-type + 125 pg eGFP mRNA (M= 241.01 µm3 SD= 36.67) versus taz-/- mutant + 125 pg eGFP mRNA (M= 146.50 µm3 SD= 21.00 p = 0.00001). Tukey’s HSD Test for multiple comparisons found that the mean value of ventricle sizes was significantly different between taz-/- mutant + eGFP mRNA (M= 146.50 µm3 SD= 21.00) versus taz-/- mutant + 100pg taz+/+ wild-type mRNA (M =188.02 µm3 SD = 41.26, p = 0.031). (B) Gene diagrams of Taz mRNA constructs, including full length taz, and various truncated taz mRNAs. (C) Embryos were injected with the indicated mRNA at the single cell stage and were fixed at 24hpf. Ventricles were visualized using atoh1a ventricle size of embryos within each group were measured, averaged, and compared amongst each other via ANOVA analysis. A one-way ANOVA revealed that there was a statistically significant difference the ventricle size between five treatment groups (F(4, 55) = [33.33], p < 0.00001). Tukey’s HSD Test for multiple comparisons found that the mean value of ventricle sizes was significantly different between taz-/- mutants injected with eGFP (M = 146.50 µm3 SD = 21.00) and Taz ΔWW (M = 75.14 µm3 SD = 30.29 p = 0.00005), e GFP and ΔPDZ (M = 217.77 µm3 SD = 37.96 p = 0.00005), but no significant differences between eGFP injected compared to Taz ΔCC (M 120.33 µm3 SD 33.55 p = 0.36) or Taz ΔTA (M 141.20 µm3 SD 39.81 p = 1.00). Representative images of embryos with ventricle visualized with atoh1a in si [file pone.0313262.s001.tif]

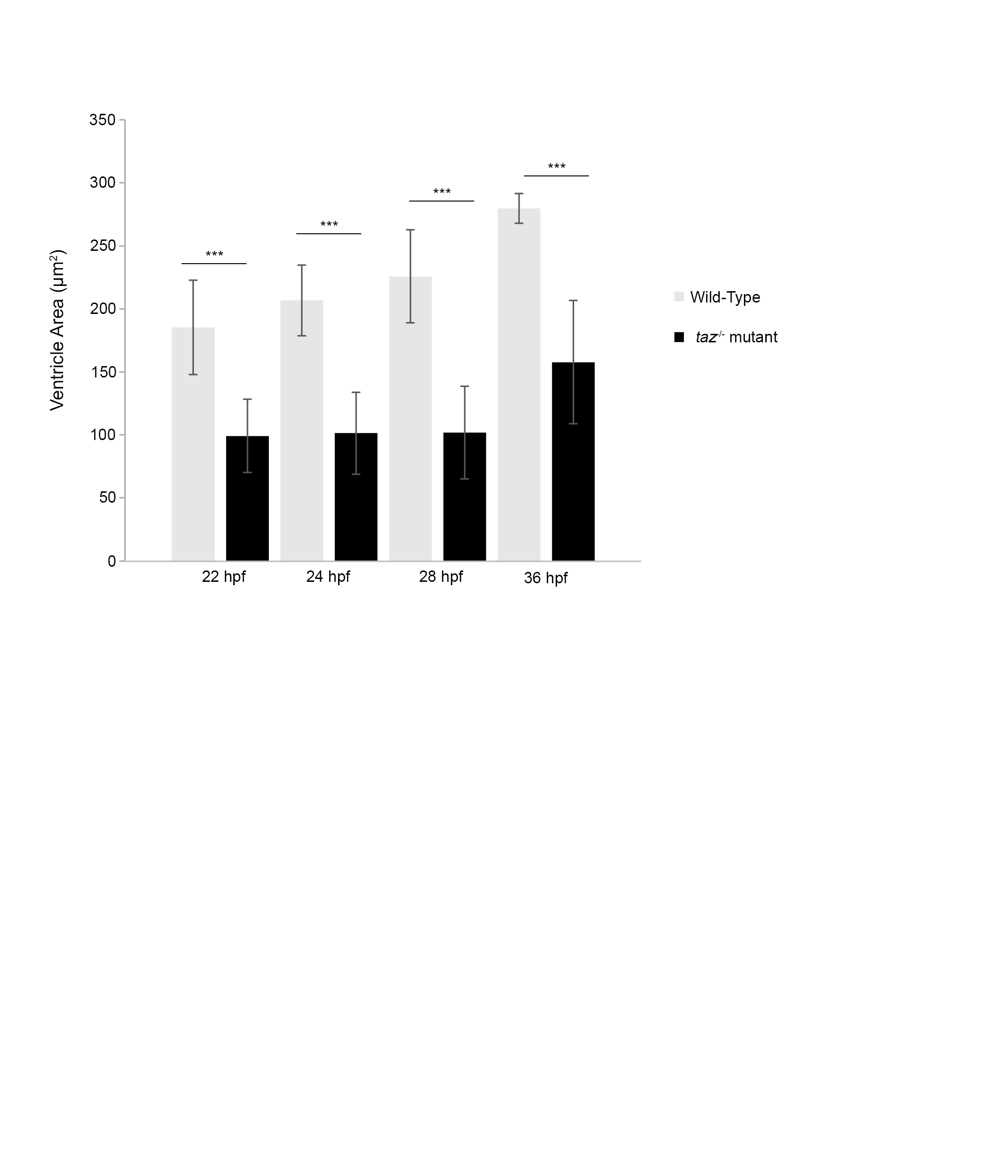

Supplement: S2 Fig — Quantification of brain ventricle size differences between wild-type (grey) and taz-/- mutants (black) after atoh1a in situ. At all stages examined (22-36 hpf) taz-/- mutants showed reduced ventricle size. 22 hpf: taz + / + wild-type M = 185.46 µM, SD = 37.58 µM versus taz-/- mutant M = 99.33 µM SD = 29.09 µM; t(19) = 5.83, p < 0.0001.; 24 hpf: taz + / + wild-type M = 206.85 µM; SD = 27.97 µM versus taz-/- mutant M = 101.60 µM; SD = 32.45 µM; t(21) = 8.29, p < 0.0001.; 28 hpf: taz+ / + wild-type M = 225.92 µM; SD = 37.00 µM versus taz mutant M = 102.01 µM; SD = 36.69 µM; t(15) = 6.63, p < 0.0001.; 36 hpf: taz + / + wild-type M = 279.69 µM; SD = 11.82 µM versus taz-/- mutant M = 157.77 µM; SD = 48.88 µM; t(19) = 5.95, p < 0.001. (TIF) [file pone.0313262.s002.tif]

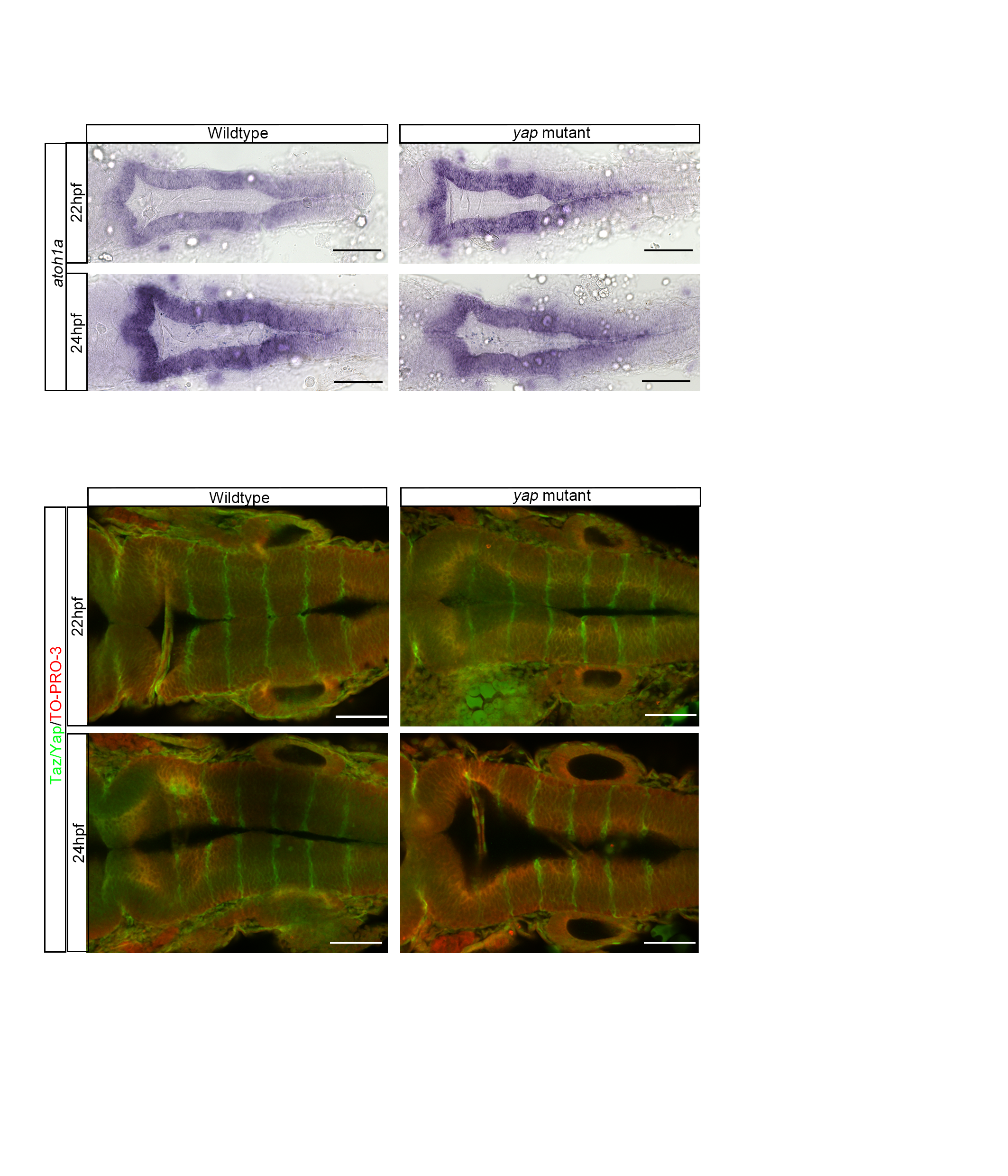

Supplement: S3 Fig — A) atoh1a in situs were performed on wildtype and yap‑/- mutants. At 22 hpf and 24 hpf, yap-/- mutant ventricles are comparable to wild-type. B) Taz/Yap immunofluorescence was performed on wildtype and yap-/- mutants. In wild-type embryos, signal is picked up within cells at the rhombomere boundaries. In yap-/- mutants, this signal at the boundaries is not diminished. Cell nuclei labelled with TO-PRO-3. Scale bars = 100 µM. (TIF) [file pone.0313262.s003.tif]

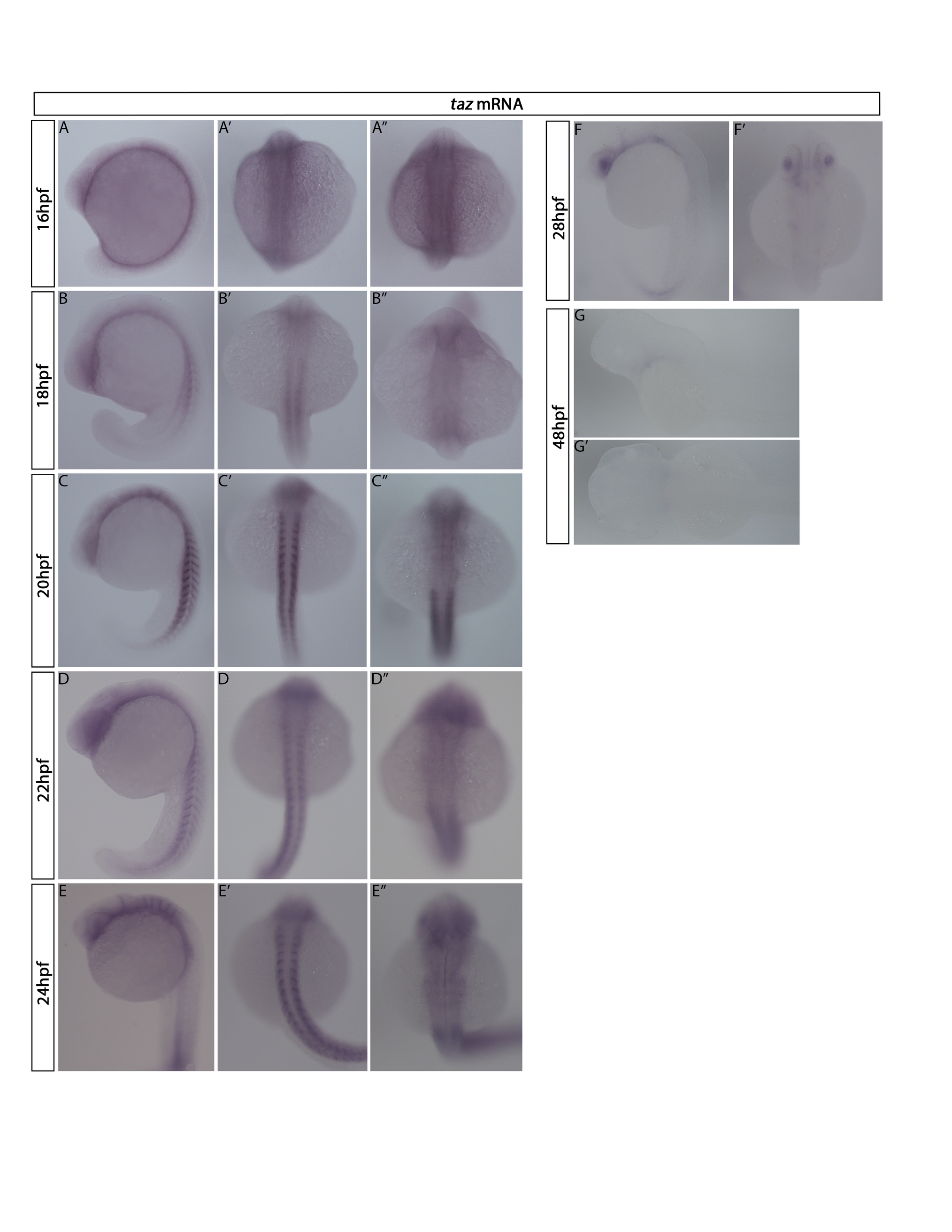

Supplement: S4 Fig — (A) in situ hybridization analyses show that taz mRNA is expressed broadly at 16 hpf with enrichment in neural tissues, shown laterally (A) and dorsally (A’, A”). (B-B”) At 18 hpf, the start of ventricle shaping, taz maintains broad expression throughout the embryo, however, at 20 hpf (C-C”) taz is enriched at rhombomere boundaries, and this enrichment persists though 22 hpf (D-D”) and to 24 hpf (E-E”). (TIF) [file pone.0313262.s004.tif]

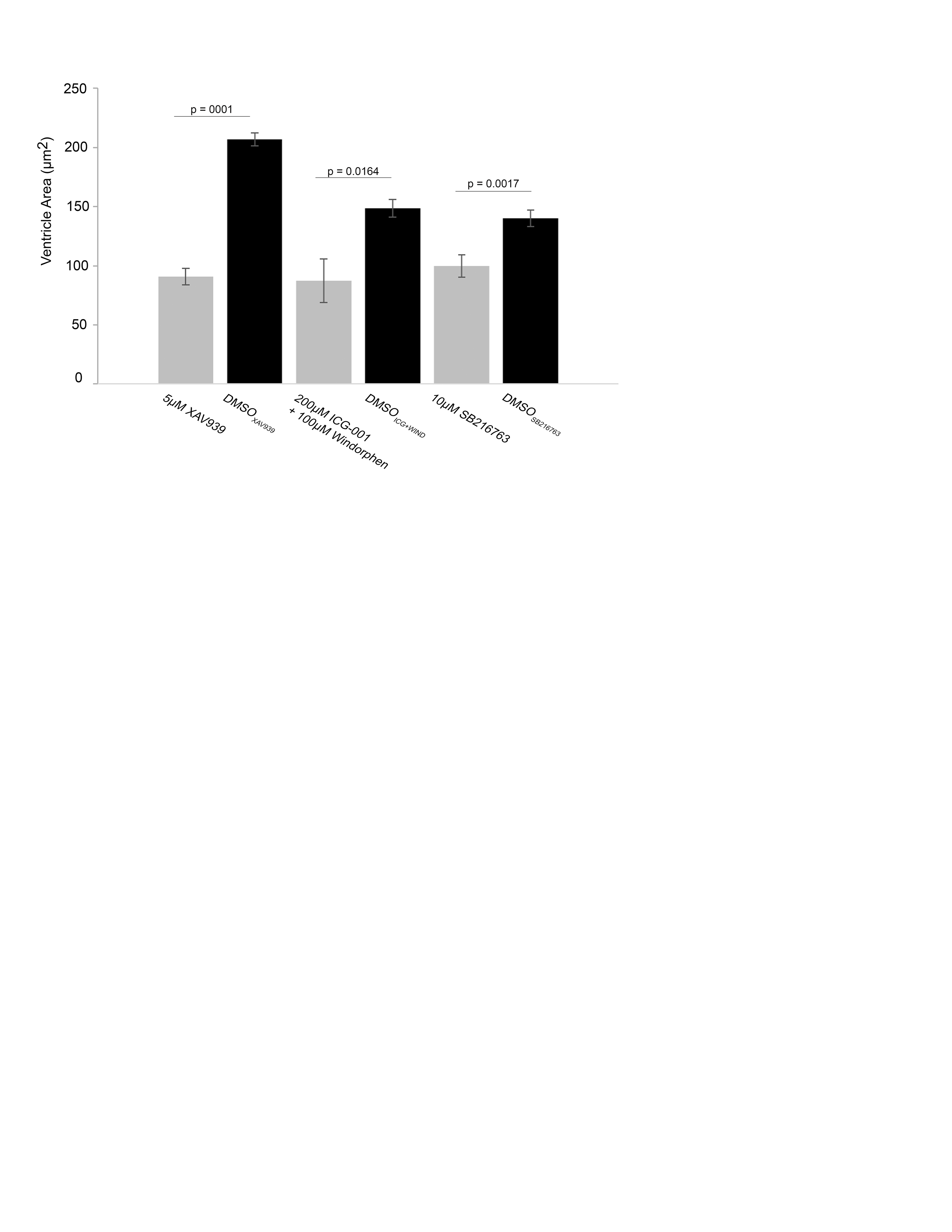

Supplement: S5 Fig — Quantification of brain ventricle size differences between DMSO controls and pharmacological manipulation of components of the Wnt pathway. XAV939 treated animals had significantly reduced ventricles (56% reduced, p-value 0.001) when compared to DMSO controls (MXAV939 = 90.69 μm3, SD XAV939 = 30.73, MDMSO = 206.95 μm3, SDDMSO = 23.05). Windorphen and ICG-001 treated animals had significantly reduced ventricles (70% reduced, p-value 0.01) when compared to DMSO controls (MWind+ICG = 87.47 μm3, SD Wind+ICG = 71.48, MDMSO = 148.48 μm3, SDDMSO = 23.87). SB216763 treated animals had significantly reduced ventricles (40% reduced, p-value 0.001) when compared to DMSO controls (MSB = 99.71 μm3, SD SB = 34.93, MDMSO = 140.21 μm3, SDDMSO = 27.58). (TIF) [file pone.0313262.s005.tif]

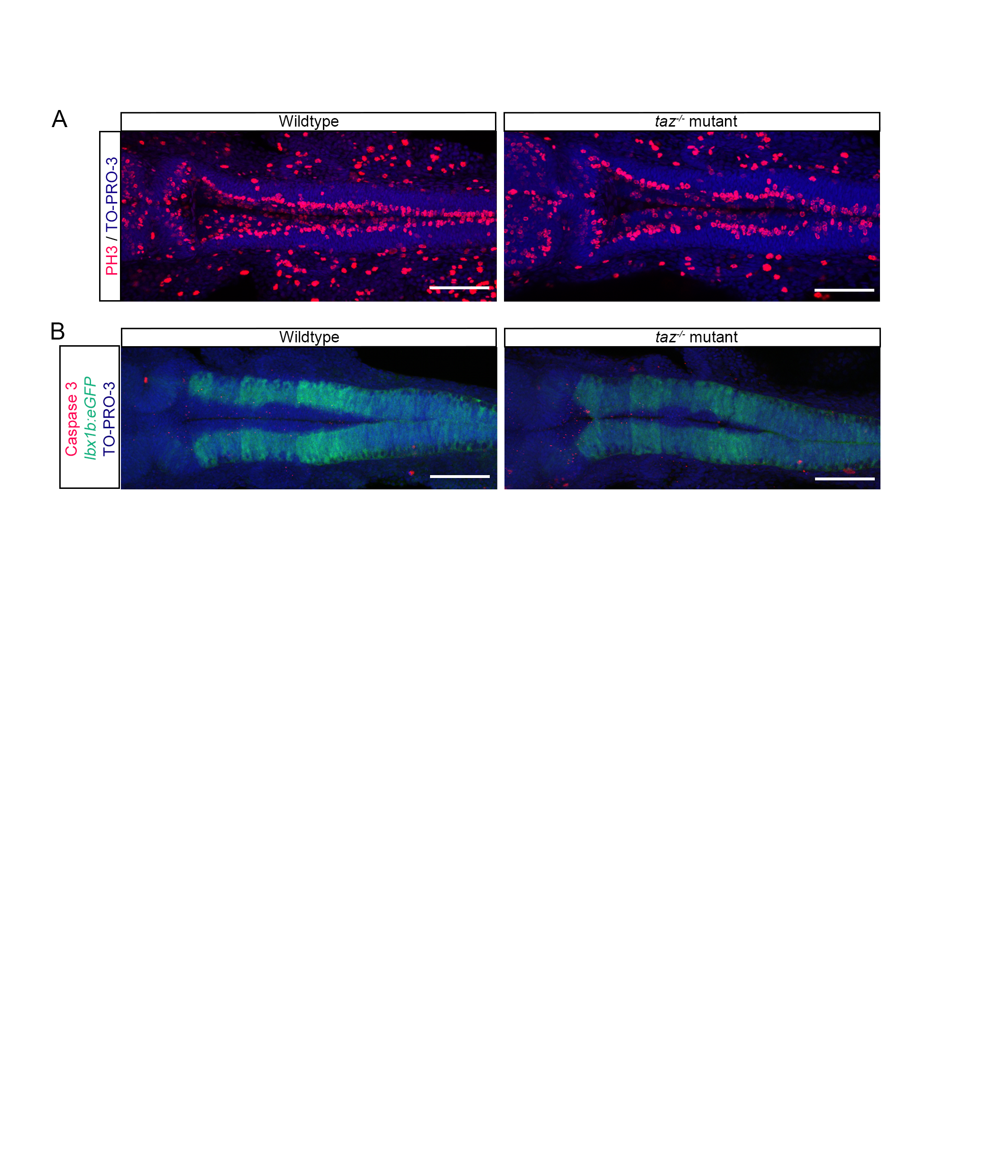

Supplement: S6 Fig — A) To assay cell proliferation, phospho-histone-H3 immunohistochemistry was performed to label actively proliferating cells. Compared to wild-type animals, taz-/- mutants show mild changes to proliferation. Cell nuclei labelled with TO-PRO-3. B) To assay cell death, cells were labelled using anti-active Caspase-3 (red). Compared to wild-type animals, taz-/- mutants show little to no changes in cell death. Tg(lbx1b:eGFP) was used to visualize the neural tube, cell nuclei labelled with TO-PRO-3. (TIF) [file pone.0313262.s006.tif]
